# Inhibition of Toll-Like Receptor 2-Mediated Interleukin-8 Production in Cystic Fibrosis Airway Epithelial Cells via the *α*7-Nicotinic Acetylcholine Receptor

**DOI:** 10.1155/2010/423241

**Published:** 2010-03-31

**Authors:** Catherine M. Greene, Hugh Ramsay, Robert J. Wells, Shane J. O'Neill, Noel G. McElvaney

**Affiliations:** Department of Medicine, RCSI Education and Research Centre, Beaumont Hospital, Dublin 9, Ireland

## Abstract

Cystic Fibrosis (CF) is an inherited disorder characterised by chronic inflammation of the airways. The lung manifestations of CF include colonization with *Pseudomonas aeruginosa* and *Staphylococcus aureus* leading to neutrophil-dominated airway inflammation and tissue damage. Inflammation in the CF lung is initiated by microbial components which activate the innate immune response via Toll-like receptors (TLRs), increasing airway epithelial cell production of proinflammatory mediators such as the neutrophil chemokine interleukin-8 (IL-8). Thus modulation of TLR function represents a therapeutic approach for CF. Nicotine is a naturally occurring plant alkaloid. Although it is negatively associated with cigarette smoking and cardiovascular damage, nicotine also has anti-inflammatory properties. Here we investigate the inhibitory capacity of nicotine against TLR2- and TLR4-induced IL-8 production by CFTE29o- airway epithelial cells, determine the role of *α*7-nAChR (nicotinic acetylcholine receptor) in these events, and provide data to support the potential use of safe nicotine analogues as anti-inflammatories for CF.

## 1. Introduction

CF is an autosomal recessive inherited disorder characterised by mutations in the gene encoding the Cystic Fibrosis Transmembrane Conductance Regulator (CFTR) protein. It is the most common inherited metabolic disorder among Caucasians of European descent, with the most common defect being the ΔF508CFTR mutation which causes the protein to fold aberrantly and accumulate in the endoplasmic reticulum of CFTR-producing cells. This leads to decreased apical expression of CFTR in airway epithelial cells, impaired Cl^−^ conductance, Na^+^ hyperabsorption, mucus hypersecretion, impaired mucociliary clearance, and colonization with microorganisms [1].

The lung manifestations of CF are characterised by chronic infection and neutrophil-dominated airway inflammation and are initiated by proinflammatory microbial stimuli culminating in increased airway epithelial cell production of proinflammatory mediators, including the neutrophil chemokine interleukin-8 (IL-8) [2]. Toll-like receptors (TLRs) play an important role in these events [3].

TLRs respond to microbial antigens and initiate signalling cascades that culminate in proinflammatory gene expression, principally via activation of the transcription factors NF*κ*B and the IRFs [4–6]. TLRs are present on a variety of cell types, including both immune cells and epithelial cells within the lung [7]. The expression and function of ten members of the human TLR family have been partially or fully characterized to date. TLRs expressed by airway epithelial cells contribute to the pulmonary immune response by regulating the production and secretion of diffusible chemotactic molecules, mucins, antimicrobial peptides, and cytokines and by enhancing cell surface adhesion molecules expression [3, 8–23]. A plethora of proinflammatory cytokines is regulated by TLR activation in airway epithelial cells; TNF*α* and IL-6 can be induced by TLR2, TLR4, and TLR9 agonists, for example, [3, 10, 21, 24]. IL-8 is a potent neutrophil chemoattractant. It is a particularly important cytokine in the neutrophil-dominated CF lung. In the context of CF and airway epithelial cells, various TLR agonists have been shown to promote proinflammatory gene transcription (reviewed in [7]). Chronic activation of TLRs can lead to overproduction of these factors and ultimately have a deleterious effect on pulmonary function and homeostasis. 

Of all the TLRs, TLR2 has emerged as the principal receptor responsible for orchestrating changes in proinflammatory gene expression in airway epithelial cells [11, 16, 17, 19, 20]. TLR2 is activated by the broadest repertoire of agonists including lipoteichoic acids, peptidoglycan, di- and tri-acylated lipopeptides from Gram-positive and/or Gram-negative bacteria, protozoans, mycobacteria, yeasts, and mycoplasma and is interesting amongst the TLR family in that it can heterodimerize with other TLRs to confer responsiveness to these diverse ligands. In conjunction with TLR1 it recognizes triacylated lipopeptides and Gram-positive lipoteichoic acid; whereas with TLR6 it can respond to diacylated lipopeptides such as MALP-2 from mycoplasma. Due to the presence of multiple potential TLR2 agonists in the CF lung, this environment represents a milieu where TLR2 is likely to be chronically activated [25]. Thus modulation of TLR2 function represents a therapeutic target for CF. 

Nicotine is a naturally occurring plant alkaloid. Although it is negatively associated with cigarette smoking, addiction, and cardiovascular damage, nicotine also has therapeutic properties and is a promising new treatment for chronic inflammatory disorders. For example nicotine is prescribed to treat the overt inflammation of gut epithelial cells in ulcerative colitis [26] and is reported to have potential therapeutic benefit for neuroinflammatory conformational disorders including Alzheimer's and Parkinson's diseases [27]. Interestingly TLRs have been shown to play a role in the disordered inflammatory response in ulcerative colitis (UC) [28].

Nicotine exerts a variety of biological effects via the nicotinic acetylcholine receptors (nAChRs), for example, inhibiting LPS-induced TNF*α*, IL-1, and IL-6 in rat peritoneal macrophages, iNOS in murine macrophages or IL-18 in human monocytes [29–31]. nAChRs are ligand-gated cation channels that comprise a pentameric transmembrane complex of multiple *α*(1-10), *β*(1-4), *γ*, *δ* or *ε* subunits, each of which has four transmembrane spanning domains that form the ion channel [32]. *α*(2-6) and *β*(2-4) can form hetero-oligomeric nAChRs, whereas *α*(7-9) subunits form homo-oligomers. It is the *α* subunit that contains the ligand binding domain. The human *α*7 subunit is ~50 kDa and is composed of 502 amino acids and a 22-residue signal peptide [32]. Studies of the anti-inflammatory effects of nicotine implicate *α*7-nAChR as the receptor involved [27, 29, 31]. The *α*7-nAChR has been shown to be present on human bronchial epithelial cells [33]. However, it remains to be determined if the *α*7 receptor is present on CF airway epithelial cells. 

In this study we investigate the effect of nicotine on IL-8 production by a CF airway epithelial cell line (CFTE29o-) in response to a range of TLR2 and TLR4 agonists. We assess expression of *α*7-nAChR in these cells and use general and specific nAChR antagonists to determine the role of *α*7-nAChR in nicotine-mediated inhibition of TLR2-induced IL-8 expression.

## 2. Materials and Methods

### 2.1. Cell Cultures and Treatments

CFTE29o- cells are a ΔF508 homozygous tracheal epithelial cell line. These were obtained as a gift from D. Gruenert (California Pacific Medical Center Research Institute, San Francisco, CA). The cells were cultured in EMEM (Invitrogen Life Technologies) supplemented with 10% foetal calf serum (FCS) at 37°C in a humidified atmosphere in 5% CO_2_. Twenty-four hours before agonist treatment, the cells were washed with serum-free EMEM and placed under serum-free conditions or in medium with 1% FCS for LPS treatments.

Stock nicotine (Sigma, 1 mg/mL or 6.2 mM in methanol) was diluted in serum-free EMEM. *Pseudomonas* LPS, peptidogylcan, zymosan, phorbol myristic acetate (PMA), d-tubocurarine, and *α*-bungarotoxin were from Sigma; triacylated lipopeptide (palmitoyl-Cys((RS)-2,3-di((palmitoyloxy)-propyl)-Ala-Gly-OH) (Pam_3_) was from Bachem.

### 2.2. IL-8 Protein Production

Cells (1 × 10^5^) were left untreated, or in some experiments pretreated with d-tubocurarine or *α*-bungarotoxin as indicated, prior to addition of nicotine at various concentrations for 1 hour at 37°C. Cells were then left untreated or stimulated with TLR2 or TLR4 agonists or PMA for 24 hours at 37°C as indicated. IL-8 protein concentrations in the cell supernatants were determined by sandwich ELISA (R & D Systems). All assays were performed in triplicate.

### 2.3. Cell Proliferation Assay

CFTE29o- cells (1 × 10^5^/mL) were left untreated or stimulated with increasing doses of nicotine (in triplicate) for 24 hours. Following this, the supernatant in each well was replaced with 500 *μ*L of serum free medium and 100 *μ*L of proliferation assay reagent (CellTiter 96 Aqueous One Solution Cell Proliferation Assay) and the samples were incubated for a further 3 hours at 37°C. Samples (120 *μ*L) were transferred from each well of the 24-well plates to a 96-well plate in duplicate. The plate was read at 490 nm. The effect of the blank well was subtracted and change in cell proliferation was measured as a percentage change from the untreated cells.

### 2.4. Laser-Scanning Cytometry

Cells (1 × 10^5^) were grown in a four-well chamber slide, washed with PBS, Fc-blocked for 15 minutes at room temperature with 1% BSA (Sigma-Aldrich), then labelled with anti-*α*7-nAChR primary antibody (Abcam) for 30 minutes at 4°C. Following three washes, cells were incubated with 10 *μ*g/mL FITC-labelled secondary antibody (antirabbit F(ab)_2_ FITC (DakoCytomation)) for 30 minutes at 4°C. Cells were counterstained with propidium iodide (PI) (Molecular Probes), and laser-scanning cytometry (LSC) (Compucyte) was used to quantify cell surface *α*7-nAChR expression. LSC is slide-based cytometry which enables the detection and quantification of cell surface expressed (or intracellular markers if a permeabilisation reagent is used) on cytospun or adherent cells without the need for trypsinization, a process which can potentially remove some receptors [3, 24, 34–40]. Cells are stained with PI enabling detection of all cell nuclei and an FITC-labelled antibody directed against the receptor of interest allows quantification of the target on the total cell population. FITC and PI cellular fluorescence of at least 2000 cells were measured. *α*7-nAChR expression was quantified using CompuCyte software on the basis of integrated green fluorescence. An appropriate rabbit antimouse isotype antibody was used as a control (DakoCytomation).

### 2.5. Statistical Analysis

Data were analysed with GraphPad Prism 4.0 software (GraphPad). Results are expressed as mean ± SE and were compared by Mann Whitney *U*-test. Differences were considered significant when the *P*-value was ≤.05.

## 3. Results

### 3.1. TLR2 and TLR4 Agonists Induce IL-8 Production from CFTE29o- Cells

**Figure 1:**
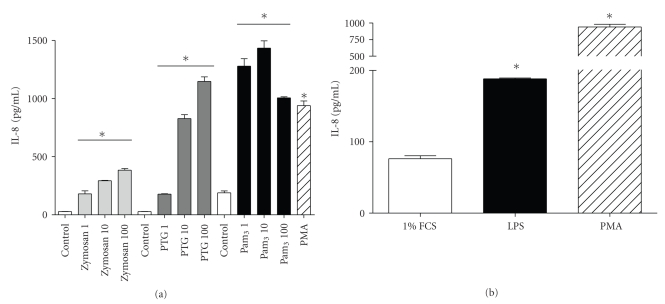
*Effect of TLR2 and TLR4 agonists on IL-8 production in CFTE29o- cells.* Triplicate samples of CFTE29o- cells (1 × 10^5^/mL) were left untreated or treated with (a) 1–100 *μ*g/mL zymosan, PTG and Pam_3_, or PMA (50 ng/mL) in serum-free media for 24 hours, or (b) *Pseudomonas* LPS (10 *μ*g/mL) or PMA (50 ng/mL) in medium supplemented with 1% FCS for 24 hours. Levels of IL-8 in supernatants were measured by ELISA and values are expressed in pg/mL (*P ≤ .05 versus control) (*n* = 7).

The effect of the TLR agonists zymosan, peptidoglycan (PTG), triacylated lipopeptide (Pam_3_), and *Pseudomonas* LPS on IL-8 production by CFTE29o- cells was quantified by ELISA (Figure 1). Each of the TLR2 agonists dose dependently increased IL-8 production by CFTE29o- cells compared to untreated cells after 24 hours treatment (Figure 1(a)). The zymosan preparation was found to be contaminated with intact yeast particles so for subsequent experiments only PTG or Pam_3,_ at 5 *μ*g/mL and 1 *μ*g/mL, respectively, were used. LPS treatment (10 *μ*g/mL, 24 hours) also significantly increased IL-8 expression by CFTE29o- cells (Figure 1(b)). PMA (50 ng/mL) is a known inducer of IL-8 and was used as a positive control.

### 3.2. Nicotine Inhibits Peptidoglycan- and Triacylated Lipopeptide-Induced IL-8 Production by CFTE29o- Cells

**Figure 2:**
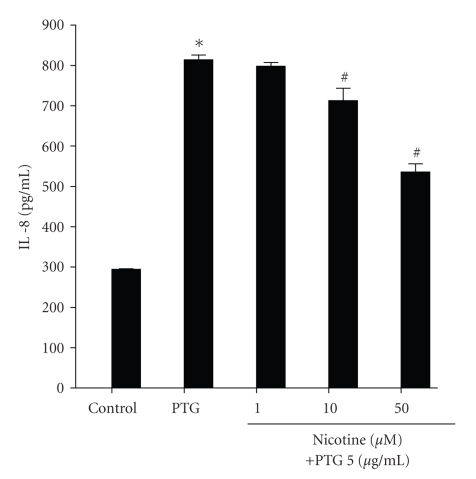
*Nicotine inhibits PTG-induced IL-8 protein expression at concentrations of 10 and 50* *μ* *M.* CFTE29o- cells (1 × 10^5^/mL) were stimulated with increasing doses of nicotine (0–50 *μ*M) for 1 hour. These samples were left untreated (control) or treated with PTG (5 *μ*g/mL, 24 hours) as indicated. Levels of IL-8 in supernatants were measured by ELISA. Assays were performed in triplicate (∗ and ^#^ *P* ≤ .05, ∗ versus control, # versus PTG) (*n* = 6).

We next investigated the effect of nicotine on TLR2 agonist-induced IL-8 production (Figure 2). As before PTG treatment (5 *μ*g/mL, 24 hours) led to a significant increase in IL-8 production from CFTE29o- cells compared to untreated controls. This response was significantly reduced in the presence of nicotine at concentrations of 10 and 50 *μ*M. The vehicle control had no effect at these doses however at a dose equivalent to 100 *μ*M nicotine, vehicle significantly impaired PTG-induced IL-8 production (data not shown). For this reason we carried out all subsequent experiments using nicotine at concentrations up to 50 *μ*M. 

**Figure 3:**
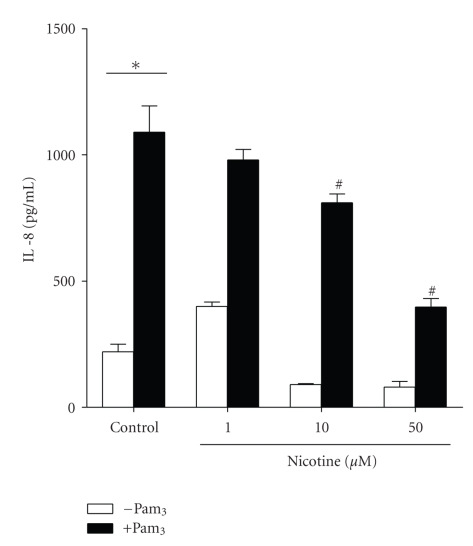
*Nicotine inhibits Pam_3_-induced IL-8 production in a dose-dependent manner.* CFTE29o- cells (1 × 10^5^/mL) were left untreated or stimulated with increasing doses of nicotine (0–50 *μ*M) for 1 hour then left untreated or stimulated with Pam_3_ (1 *μ*g/mL, 24 hours) as indicated. Levels of IL-8 in supernatants were measured by ELISA and values are expressed in pg/mL (∗ and ^#^ *P* ≤ .05, ∗ versus control, # versus Pam_3_). Assays were performed in triplicate (*n* = 5).

Figure 3 shows that nicotine also significantly inhibited Pam_3_-induced IL-8 expression from CFTE29o- cells at 10 and 50 *μ*M.

### 3.3. Nicotine Does Not Inhibit LPS-Induced IL-8 Production by CFTE29o- Cells

**Figure 4:**
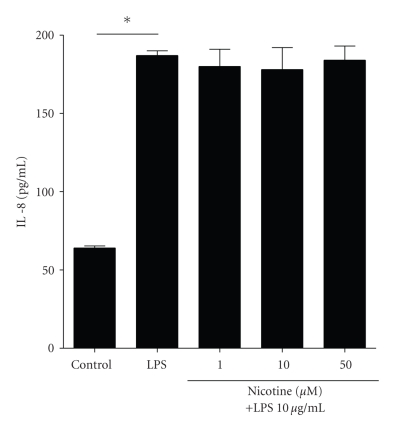
*No effect of nicotine on LPS-induced IL-8 protein production in CFTE29o- cells.* CFTE29o- cells (1 × 10^5^/mL) were left untreated or stimulated with increasing doses of nicotine (0–50 *μ*M) for 1 hour. Following this, samples were either left untreated or treated with *Pseudomonas* LPS (10 *μ*g/mL, 24 hours). Levels of IL-8 in supernatants were measured by ELISA and values are expressed in pg/mL (*P ≤ .05). Assay was performed in triplicate (*n* = 4).

Next the effect of nicotine on IL-8 production induced by the TLR4 agonist *Pseudomonas* LPS was assessed. These assays were performed in the presence of 1% FCS to facilitate LPS-TLR4 signalling. Figure 4 shows that LPS-induced IL-8 production was not significantly inhibited by pretreatment with nicotine at concentrations of 1–50 *μ*M.

### 3.4. Effect of Nicotine on CFTE29o- Proliferation

**Figure 5:**
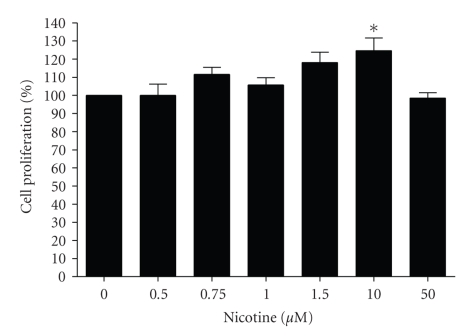
*Nicotine does not increase CFTE cell death but increases cell survival at certain concentrations.* CFTE29o- cells (1 × 10^5^/mL) were left untreated or stimulated with increasing doses of nicotine (in triplicate) for 24 hours. Cell proliferation was quantified and values are expressed as a percentage change from the untreated cells (*P ≤ .05).

Nicotine has known antiapoptotic effects in a variety of cells [41–44]. However in order to determine that nicotine's ability to decrease TLR2-induced IL-8 production was not being mediated by increased cell death or apoptosis, the effect of nicotine on CFTE29o- cell proliferation was tested. Figure 5 shows that over a range of concentrations up to 50 *μ*M, nicotine was nontoxic to CFTE29o- cells and at 10 *μ*M nicotine has a significant protective effect and actually promoted cell survival (*P = .0286).

### 3.5. CFTE29o- Cells Express the *α*7-nAChR

**Figure 6:**
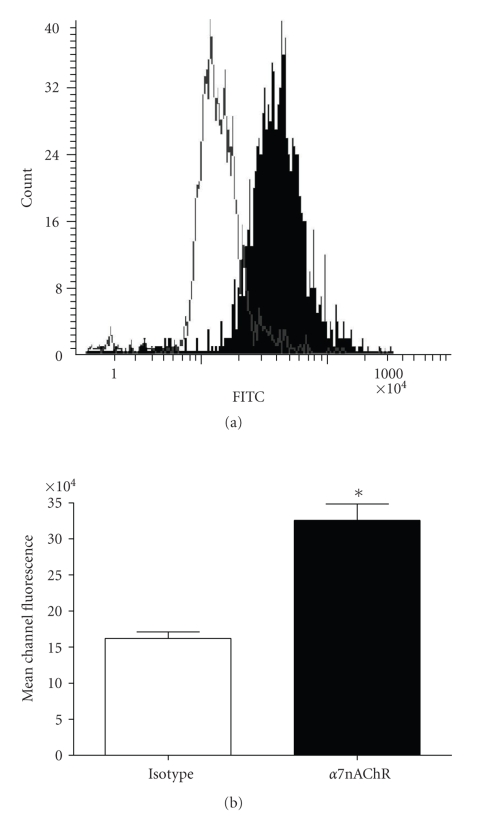
*CFTE29o- cells express the *α*7-nAChR.* CFTE29o- cells (1 × 10^5^/mL) were grown in chamber slides, Fc-blocked, and labelled with FITC-conjugated anti-*α*7-nAChR or isotype control antibodies. Cells were counterstained with PI, and *α*7-nAChR surface expression was quantified by laser-scanning microscopy. (a) Representative histogram of FITC fluorescence comparing isotype (clear) and anti-*α*7-nAChR (solid) antibody-labelled samples. (b) Histogram showing median channel fluorescence ± SEM (*P ≤ .05,  *n* = 6).

Nicotine is known to exert an anti-inflammatory effect through the *α*7-nAChR [45]. We used laser scanning microscopy to examine cell surface expression of *α*7-nAChR on CFTE29o- cells. Figure 6 illustrates that CFTE29o- cells express the *α*7-nAChR; the histogram in Figure 6(a) shows clear detection of *α*7-nAChR with an anti-*α*7-nAChR antibody (solid) compared to an isotype control antibody (clear). In Figure 6(b) the median channel fluorescence (MCF) emitted by the FITC-linked anti-*α*7-nAChR antibody is significantly greater than that of the isotype antibody (163,710 ± 31,788 versus 325,680 ± 55,554 MCF, *P* = .0011).

### 3.6. *α*7-nAChR Mediates Nicotine's Inhibitory Effect on TLR2-Induced IL-8 Production in CFTE29o- Cells

**Figure 7:**
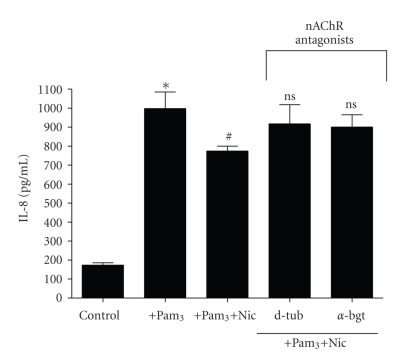
*Inhibition of *α*7-nAChR abrogates nicotine's anti-TLR2 inhibitory effect.* CFTE29o- cells (1 × 10^5^/mL) were left untreated or treated d-tubocurarine (d-tub, 100 *μ*M) or *α*-bungarotoxin (*α*-bgt, 1 *μ*M) for 16 h. Nicotine was added as indicated at 10 *μ*M for 1 hour. Following this, some samples were stimulated with Pam_3_ (1 *μ*g/mL, 24 hours) and levels of IL-8 in supernatants were measured by ELISA (∗ and ^#^ *P* ≤ .05, ∗ versus control, # or ns versus Pam_3_). Assays were performed in triplicate (*n* = 3).

Finally we investigated whether nicotine mediates its anti-inflammatory effects via *α*7-nAChR in CF airway epithelial cells. To do this we employed the use of d-tubocurarine, a broad-range nAChR inhibitor, and *α*-bungarotoxin, a specific *α*7-nAChR inhibitor. For these experiments we used nicotine at 10 *μ*M and as before this dose significantly inhibited Pam_3_-induced IL-8 protein production (Figure 7). Pretreatment with either antagonist for 1 hour had no effect on nicotine's ability to inhibit the TLR2 response (data not shown). However pretreatment for 16 h with the broad range nAChR antagonist d-tubocurarine reversed the inhibitory effect of nicotine on Pam_3_-induced IL-8 expression, with IL-8 levels not significantly different from those induced by Pam_3_ alone. Similarly 16 h pretreatment of CFTE29o- cells with *α*-bungarotoxin (1 *μ*M) abrogated nicotine's ability to decrease expression of IL-8 in response to Pam_3_. These data implicate *α*7-nAChR in nicotine's anti-TLR2 effect.

## 4. Discussion

Whilst inflammation in the CF lung is a neutrophil-dominated process, the airway epithelium plays a key role in the regulation of neutrophil recruitment via TLR-mediated changes in gene and protein expression [3]. Here we show that CF airway epithelial cells express *α*7-nAChR and respond to nicotine by inhibiting TLR2 agonist-induced IL-8 expression. This novel finding is of particular interest with respect to CF, as the CF lung is a milieu rich in potential TLR2 agonists and because TLR2 is the predominant TLR expressed on the surface of lung epithelial cells in vivo [11, 16, 17, 19, 20, 25]. 

The mechanism by which nicotine can exert its anti-inflammatory effects has been reported to include targeting NF*κ*B and AP1 [46, 47]; the IL-8 gene is regulated by both of these transcription factors. For example, nicotine in cigarette smoke extract can inhibit transcription of LPS-induced IL-1, IL-8, and PGE_2_ in activated macrophages through inhibition of the NF*κ*B pathway. Although we did not observe inhibition of LPS-induced IL-8 expression in CF airway epithelial cells, others have reported that nicotine can inhibit LPS-induced NF*κ*B DNA binding and transcriptional activity. Indeed several studies have linked the anti-inflammatory function of nAChRs to the NF*κ*B pathway [46, 48–53]. Yoshikawa et al. [54] further reported that the mechanism by which nicotine impairs NF*κ*B activation in human peripheral monocytes is via inhibition of phosphorylation of I*κ*B. Given that TLR4 and TLR2 share the same signalling pathways, it is likely that nicotine also inhibits TLR2-induced IL-8 expression by targeting NF*κ*B and possibly AP1 [4–6]. 

The anti-inflammatory effects of nicotine can be mediated via *α*7-nAChR [45], and our studies clearly implicate *α*7-nAChR in nicotine's anti-TLR2 activity in CF airway epithelial cells. A range of nAChRs has been shown to be present on human epithelial cells, including *α*7-, *α*3-, and *α*3*β*4-subtypes [33, 55]. Normal bronchial epithelial cells express *α*7-nAChR. Our studies have detected *α*7-nAChR on CF tracheal epithelial cells for the first time and show that specific inhibition of *α*7-nAChR using *α*- bungarotoxin (a 75 amino acid peptide from *Bungarus multicinctus* venom) abrogates nicotine's ability to impair Pam_3_-induced IL-8 protein production. Thus *α*7-nAChR may represent a new therapeutic target for CF. Agonists of *α*7-nAChR have previously been proposed for the treatment of inflammatory diseases via their ability to reduce TNF*α* release from macrophages. For example in vivo treatment with nicotine can inhibit TNF*α*-induced HMGB1 secretion and has a proven therapeutic benefit in models of sepsis [48]. In these studies nicotine did not affect levels of total or phosphorylated versions of ERK, JNK, or p38 MAPK, rather the observed effects occurred directly via *α*7-nAChR-mediated blockade of NF*κ*B. 

A major drawback to the potential use of nicotine as a therapeutic agent is its negative side effects which are associated with addiction, cardiovascular disease, hypertension, cancer, reproductive and gastrointestinal disorders. However, nicotine analogues exist that lack addictive or damaging side effects but retain desirable anti-inflammatory and cognitive-enhancing properties. Indeed the objective in developing nicotine analogues is the discovery of novel drugs that feature the beneficial actions of nicotine whilst eschewing its side-effect profile [56, 57]. The addictive properties of nicotine are mediated via the *β*2-containing nAChR subtypes, hence compounds that are selective for the *α*7-nAChR—the receptor that mediates nicotine's anti-inflammatory effects—are attractive as potential therapeutic agents. Varenicline is a partial agonist of the *α*_4_ *β*_2_ receptor and a full agonist of *α*7-nAChR that is currently used as a smoking-cessation therapy. Given its nAChR affinity, unlike nicotine, it lacks addictive effects but retains anti-inflammatory benefits [58]. Thus evaluation of the anti-inflammatory properties of varenicline for CF would be worthy of further study.

Notwithstanding the novelty of this study the observations are limited somewhat by the fact that only a single CF epithelial cell line was used, cytokines other than IL-8 were not measured and nicotine analogues were not tested. It will also be important to explore in greater detail the mechanism by which nicotine achieves its anti-inflammatory effect in CF epithelium. These questions will form the basis of future studies. 

In conclusion the findings of this study indicate that nicotine and nicotine analogues have potential to inhibit TLR2-mediated inflammation in response to common agonists in the CF lung via *α*7-nAChR. These useful effects occur at dose levels that could be delivered to CF lungs through inhaled preparations.
